# RC-4BC cells express nicotinic and muscarinic acetylcholine receptors

**DOI:** 10.1371/journal.pone.0279284

**Published:** 2022-12-16

**Authors:** Gulsamal Zhubanova, Olena Filchakova

**Affiliations:** Biology Department, School of Sciences and Humanities, Nazarbayev University, Nur-Sultan, Republic of Kazakhstan; Weizmann Institute of Science, ISRAEL

## Abstract

Acetylcholine is one of the most important endogenous neurotransmitters in a range of organisms spanning different animal phyla. Within pituitary gland it acts as autocrine and paracrine signal. In a current study we assessed expression profile of the different subunits of nicotinic as well as muscarinic acetylcholine receptors in RC-4BC cells, which are derived from rat pituitary gland tumor. Our findings indicate that β2, δ, and M2 subunits are expressed by the cells with the lowest Ct values compared to other tested subunits. The detected Ct values were 26.6±0.16, 27.95±0.5, and 28.8±0.25 for β2, δ, and M2 subunits, respectively.

## Introduction

Endogenous neurotransmitter acetylcholine (ACh) acts on nicotinic receptors as well as on muscarinic receptors. Nicotinic acetylcholine receptors (nAChRs) are pentameric ligand-gated ion channels, with diverse structure and function. Each subunit bypasses plasma membrane four times, has extracellular N- and C-termini, with ligand-binding site at the N-terminus [1]. There are seventeen subunits characterized in vertebrates [2]. They include muscle-type α1, β1, δ, ε, and γ subunits. Functional receptor in muscle cells is composed of two α1 subunits, β1, δ, and either γ or ε subunits Besides muscle-type receptor subunits, neuronal subunits include α2, α3, α4, α5, α6, α7, α9, α10, and β2, β3, β4. The neuronal receptors can assemble into homomeric or heteromeric structures. Homomeric receptors are composed of a single type of α subunit, such as in α7 receptor. Heteromeric receptors contain α subunits combined with β subunits in different stoichiometries. Neuronal receptors are widely distributed within central nervous system (CNS), as well as outside of it [3].

Muscarinic acetylcholine receptors (mAChRs) are seven-transmembrane domain G-protein coupled receptors. There are five receptor subtypes identified, named M1 –M5. M1, M3, and M5 receptors are coupled to G_q/11_, with activation of phospholipase C [4]. Activation of M2 and M4 receptors lead to inhibition of adenylate cyclase, and downregulation of cAMP.

Pituitary gland is divided into two functional parts: anterior and posterior pituitary. While posterior pituitary releases hormones into the bloodstream directly, being a continuation of hypothalamus, anterior pituitary is connected with hypothalamus via network of blood vessels, and itself is a gland that contains multiple types of cells releasing multitude of hormones into the bloodstream. The cells within anterior pituitary are specialized into five endocrine cell types differing by the secreted hormone. These cell types include gonadotrophs releasing luteinizing hormone (LH) and follicle stimulating hormone (FSH), thyrotrophs releasing thyroid stimulating hormone (TSH), somatotrophs producing growth hormone (GH), corticotrophs releasing adrenocorticotropic hormone (ACTH), and lactotrophs secreting prolactin. Within anterior pituitary there are also non-endocrine folliculostellate cells, which have glial cell morphology, they affect secretion of pituitary hormones [5]. ACh is synthesized by hypothalamus [6, 7]. It acts in autocrine/paracrine signaling, as well as in endocrine signaling being released into the hypophyseal portal system. Early binding studies suggested functional muscarinic receptors within rat pituitary [8–10]. Nakajima et al. [11] showed that Ach increases intracellular Ca2+ within folliculostellate cells in rat primary cell culture. The same group suggested that M1 muscarinic receptors were involved as far as Ca2+ rise was sensitive to atropine and pirenzepine, and to phospholipase C inhibitors. *In situ* hybridization study demonstrated expression of α9 nAChR subunit in pars tuberalis of rat pituitary [12]. α10 subunit was also demonstrated within pars tuberalis of pituitary [13]. In the current study we aimed at identifying different types of nicotinic as well as muscarinic acetylcholine receptor subunits in pituitary cells. We conducted our experiment on RC-4BC cell like, as far as this cell line is derived from aged rat pituitary adenoma [14], and it contains different types of cells similar to the cell types within anterior pituitary.

## Methods and materials

### Cells and reagents

RC-4B/C cell line was obtained from the American Type Culture Collection (ATCC CRL-1903). The cells were maintained at 37°C in a humidified atmosphere with 5% CO_2_ and were cultured in complete medium which contained Dulbecco’s modified Eagle’s medium with 4 mM L-glutamine adjusted to contain 1.5 g/L sodium bicarbonate and 4.5 g/L glucose, 45%; alpha minimum essential medium with 1 g/L glucose, 45%; supplemented with the 0.01 mM nonessential amino acids, 15 mM HEPES, 0.2 mg/ml bovine serum albumin, 2.5 ng/ml epidermal growth factor, dialyzed heat-inactivated fetal bovine serum, 10% and penicillin-streptomycin solution (with 10,000 units of penicillin and 10 mg/ml of streptomycin), diluted 100x. The complete medium was refreshed every 2 days. Cells used for the experiment were under tenth passages.

### RNA isolation, cDNA synthesis and PCR

Total RNA was isolated from RC-4B/C cell line using RNeasy Mini Kit (Qiagen, 74104), according to the manufacturer’s protocol. cDNA was synthesized using the Maxima First Strand cDNA Synthesis Kit for RT-qPCR, with dsDNase (ThermoFisher, K1672). 1 μg of RNA was used for the synthesis, synthesis was according to the manufacturer’s protocol. Afterwards, amplification of target fragments was carried out on a PCR thermocycler. 20 μl of PCR reaction included 2 μl template DNA, 1 μl forward primer (10 pmoles), 1 μl reverse primer (10 pmoles), 2 μl dNTP, 2 μl 10x buffer, 0.25 μl Taq DNA polymerase (5U/μL, NEB, M0273X), 11.75 μl nuclease-free H_2_O. The PCR reaction conditions were initial denaturation at 95°C for 3 min, 30 cycles of denaturation at 95°C for 30 s, annealing at 55°C for 30 s, extension at 72°C for 30 s, followed by final extension at 72°C for 7 min. Bands were detected by 1.5% agarose gel electrophoresis. As ladder DirectLoad PCR 100 bp Low Ladder (D3687, Sigma-Aldrich) was used. After electrophoresis, sample was analyzed on GelDoc.

### RT-qPCR

For RT-qPCR using nAChR and mAChR primers, all polymerase chain reaction primers were purchased from Lumiprobe RUS Ltd. (Moscow, Russian Federation) (Table 1). qPCR was performed and analyzed on CFX96 Touch Real-Time PCR Detection System to determine the relative amounts of α1, α2, α3, α4, α5, α6, α7, α9, α10, β1, β2, β3, β4, δ, ε nAChR subunits and M1, M2, M3, M4, and M5 mAChRs at mRNA level. SsoAdvanced Universal SYBR Green Supermix (Bio-Rad, 1725271) was used for qPCR reactions. PCR program was as follows: initial denaturation at 95°C, followed by 30 cycles of denaturation at 95°C for 10 sec and annealing at 60°C for 30 sec, followed by melt curve analysis at 55–95°C in 0.5°C increments with 4 sec/step. Three technical replicates were used. The experiment was repeated in duplicates. Ct values are number of cycles required to cross fluorescence threshold value, which is set up automatically.

**Table 1:** Primers used in the study.

Gene	GenBank accession number	Primer	PCR product (bp)
**CHRNA1**	NM_024485.2	Forward: 5’-CGGGAAGTACATGTTGTTTACC-3’	180
		Reverse: 5’-CTGGATGGTCTTTTCATTGTGG-3’	
**CHRNA2**	NM_133420.1	Forward: 5’-GCTCTTCACCATGATCTTTGTC-3’	235
		Reverse: 5’-AGCATCCATGTTAGTCTCTAGC-3’	
**CHRNA3**	NM_052805.2	Forward: 5’-TGAAGGTGGATGAAGTAAACCA-3’	165
		Reverse: 5’-CGTTGTTGTACAGTACGATGTC-3’	
**CHRNA4**	NM_024354.2	Forward: 5’-GTACCTCCTCTTCACCATGATC-3’	277
		Reverse: 5’-GTTGCAGATGTCACTCAAGATG-3’	
**CHRNA5**	NM_017078.2	Forward: 5’-CGTACTCCTTTGTGATTAAGCG-3’	267
		Reverse: 5’-CCATAATGGATAGGGTCACGAA-3’	
**CHRNA6**	NM_057184.2	Forward: 5’-AAGTGGACATGAACGACTTTTG-3’	153
		Reverse: 5’-TGGTATAAAACATGGGCAGTCT-3’	
**CHRNA7**	NM_012832.3	Forward: 5’-CGGAGTGAAGAATGTTCGTTTT-3’	154
		Reverse: 5’-GAATATGCCTGGAGGGAGATAC-3’	
**CHRNA9**	NM_022930.2	Forward: 5’-GGTTGCGTATGCTTTTATTCCT-3’	204
		Reverse: 5’-CACTGTGCTTTGTTGTCTACAA-3’	
**CHRNA10**	NM_022639.1	Forward: 5’-TTGATATGGATGAACGGAACCA-3’	159
		Reverse: 5’-TGTAAAGTACGATGTCTGGTCG-3’	
**CHRNB1**	NM_012528.1	Forward: 5’-TTATGATAGCTCAGTAAGGCCG-3’	112
		Reverse: 5’-GCTCATTTCTTCATCCTTCTCG-3’	
**CHRNB2**	NM_019297.2	Forward: 5’-GACAATATGAAGAAAGTCCGGC-3’	115
		Reverse: 5’-AGACCACAGCATTGGAATAGAA-3’	
**CHRNB3**	NM_133597.2	Forward: 5’-GTTCTCTAAGGCAGGTGTTACT-3’	149
		Reverse: 5’-TACAGTTCTACTAGCGAATGGC-3’	
**CHRNB4**	NM_052806.2	Forward: 5’-CTATGACTTCATCATCAAGCGC-3’	242
		Reverse: 5’-CCATGGTGAACAAGAGGTACTT-3’	
**CHRND**	NM_019298.1	Forward: 5’-CATTCCAGATTTCTTACGCCTG-3’	153
		Reverse: 5’-TGAGTGAACTGAATTTGAGGGA-3’	
**CHRNE**	NM_017194.1	Forward: 5’-GAGGAGCTCATCTTGAAAAAGC-3’	239
		Reverse: 5’-CAAAAACAGACATTGTCGAGGG-3’	
**CHRM1**	NM_080773.1	Forward: 5’-CTCACCTGGACACCATATAACA-3’	104
		Reverse: 5’-TTGACGTAGCATAGCCAGTAG-3’	
**CHRM2**	NM_031016.2	Forward: 5’-CAATGTCATGGTGCTCATCAAT-3’	208
		Reverse: 5’-CTTTTGATGGTCTTTTCACCGT-3’	
**CHRM3**	NM_012527.2	Forward: 5’-TCCCTGATGGTGATAAAATGGG-3’	144
		Reverse: 5’-CACATCTCTTCATGTTTAGCGG-3’	
**CHRM4**	NM_031547.1	Forward: 5’-CATCTGCTGAAGAGAACAGGTC-3’	80
		Reverse: 5’-TCAGTAGAGATCTCTCCCATCC-3’	
**CHRM5**	NM_017362.5	Forward: 5’-GCTCAGATCTTTCTTTAGCTGC-3’	108
		Reverse: 5’-GTTGTCTTTCCTGTTGTTGAGG-3’	

## Results

In order to evaluate the expression of the acetylcholine receptors in RC-4BC cells, RT-PCR approach were used. The sequence of primers and expected product size are presented in Table 1.

**Fig 1:**
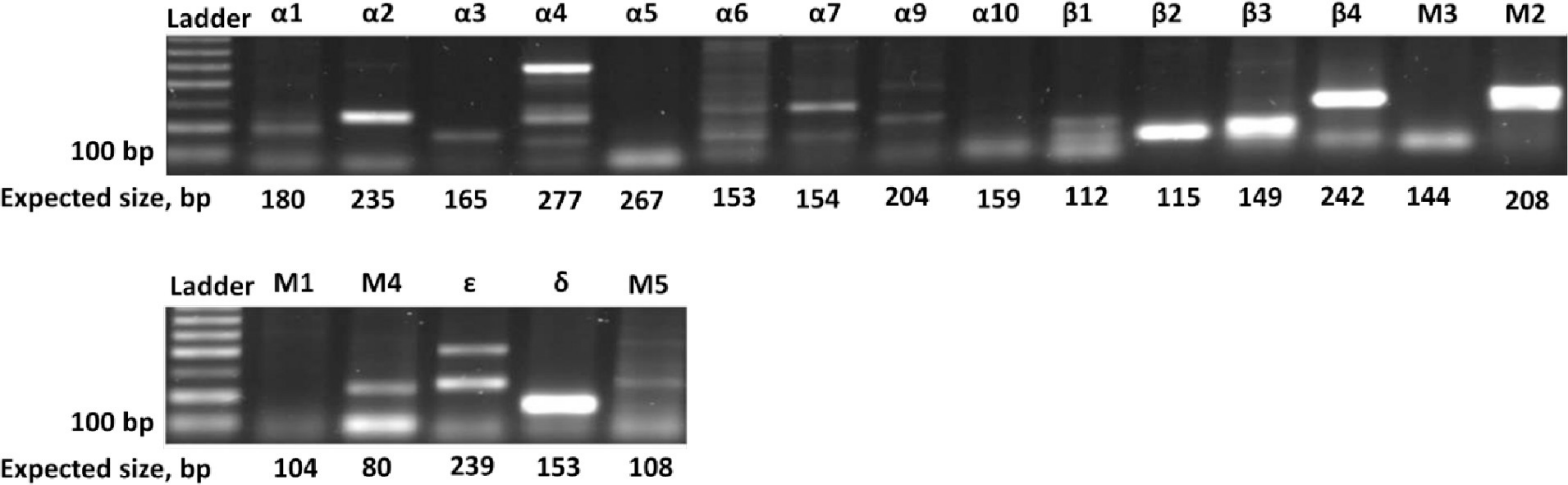
Representative data of expression of different receptor subunits in RC-4BC cells. 100 bp ladder was used. The expected sizes of PCR products are shown.

From the initial expression data amplicons of expected size were detected for α2 and δ subunits (Fig 1). In addition, bands were observed for β2, β3, β4, M2, and M3 subunits. Alongside the expected bands of appropriate size, multiple bands were observed for α4, M4, and ε subunits. Not conclusive bands were observed for α1, α3, α5, α6, α7, α9, α10, β1, M1, and M5 subunits.

**Fig 2:**
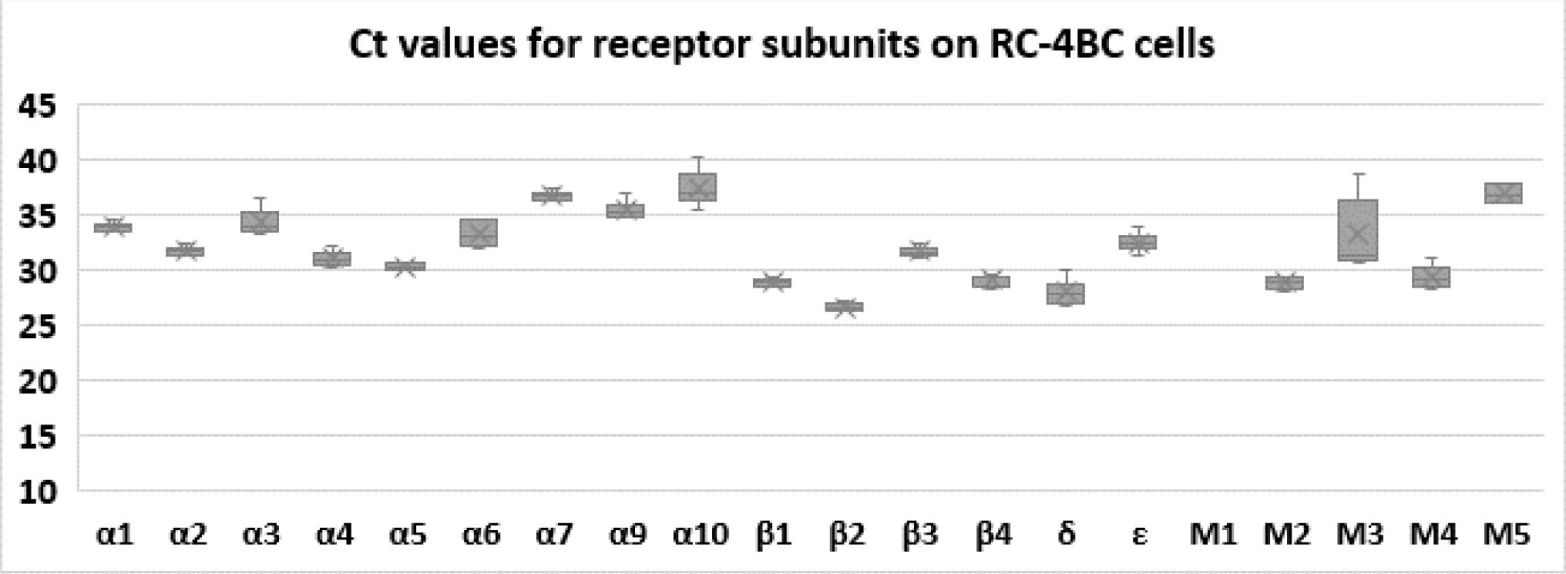
Ct values for each subunit tested. The mean Ct values for each subunits with SEM were as follows: α1–33.9±0.16 (n = 6), α2–31.7±0.15 (n = 6), α3–34.4±0.47 (n = 6), α4–31.0±0.29 (n = 6), α5–30.3±0.12 (n = 6), α6–33.3±0.45 (n = 6), α7–36.8±0.17 (n = 6), α9–35.5±0.33 (n = 6), α10–37.4±0.67 (n = 6); β1–28.9±0.13 (n = 6), β2–26.6±0.16 (n = 6), β3–31.65±0.19 (n = 6), β4–29.05±0.22 (n = 6); δ– 27.95±0.5 (n = 6), ε– 32.5±0.35 (n = 6); M1 –not amplified, M2–28.8±0.24 (n = 6), M3–33.2±1.5 (n = 5), M4–29.3±0.46 (n = 6), M5–36.9±0.5 (n = 3). N corresponds to the number of wells pooled from two experiments.

The expression of the subunits was checked by RT-qPCR, and following results for Ct values were obtained (Fig 2). Three technical replicates were used per reaction. The lowest Ct value was observed for β2 subunit (26.6±0.16, n = 6), followed by δ subunit (27.95±0.5, n = 6), where n represents the number of wells pooled from independent experiments. β1, β4, and M4 subunits had Ct values below 30. M1 subunit was at non-detectable level, while α7, α9, α10 subunit, as well as M5 receptor each had Ct values higher than 35.

## Discussion

RC-4BC cell line was derived from an aged male rat pituitary adenoma. It contains all types of anterior pituitary cells with majority of cells containing FSH, LH, and prolactin, with many bihormonal cells within cell line [15]. The cell line was used to study basal prolactin secretion in comparison with primary pituitary cells and other aspects of pituitary cell functionality [16, 17]. They are suitable to address questions related to pituitary adenoma growth. RC-4BC cells were used to create 3D model of pituitary tumor [18]. The cells were used to address the regulatory role of miR-29-3p [19] and miR-410-3p [20] on cell proliferation. Additionally, the cells can be used to study binding affinities of different ligands, as was done for prolactin-releasing peptide and its palmitoylated analog [21]. RC-4BC can be used to study antiproliferative potential of drugs for treatment of pituitary adenomas [22]. In the current study we aimed to investigate the expression of different subunits of acetylcholine receptors.

In the current study we detect high expression level of β2 subunit. β2 subunit constitutes part of heteromeric nAChRs. It is worth investigation, whether β2 subunit contributes to the formation of functional receptors within studied cells. Previously it was shown that rat gonadotrophs express functional β2-containing nicotinic receptors (23). α4β2 subunit is the most prevalent receptor type within brain [23]. Our results for β2 subunit expression coincide with finding by Zemkova et al. [24], who demonstrated the highest level of expression for β2 subunit in rat primary culture cells compared to other nicotinic receptor subunits.

Our study suggests that α7 and α9 subunits of homomeric receptor subtypes are expressed at low level within RC-4BC cells. It is quite interesting considering that α7 receptor is abundantly present within hippocampal region [25], while α9 receptor is present in rodent pituitary gland within pars tuberalis [12]. Zemkova et al. also demonstrated expression of α9 subunit within rat pituitary cells [24]. The discrepancy in findings could be explained by different nature of tested cells: RC-4BC cells as in our case and primary cells as in previous studies. The discrepancy in the expressed receptors between primary cell culture and immortalized cells was noted before [7]. The cells within cell line over multiple passages could potentially change the expression profile of receptor subunits. RC-4BC cell line is a heterogeneous cell line that contains different types of pituitary cells. Further studies are needed to dissect the expression of nicotinic receptor subunits within different cells of such heterogeneous cell mixture.

δ subunit amplified robustly in our experiment. This is quite unexpected as far as δ subunit constitutes the structural subunit of muscle-type nicotinic acetylcholine receptor, and mutations within this particular receptor subunit result in congenital myasthenic disease [26]. While being essentially a muscle-type of receptor subunit, there are studies indicating sole expression of δ subunit in particular cell line. For example, it is expressed in human cerebellar medulloblastoma cell line TE671 [27].

M2 subunit robustly amplified in our study. Zemkova et al. [24] that showed the expression of M4 subunit in rat pituitary cells as well as in immortalized LβT2 gonadotrophs. Both M2 and M4 receptors function through inhibition of adenylate cyclase and downregulation of the level of cAMP within the cell [28]. M2 receptor is abundantly present within hearth muscle cells, where its activation affects the contractibility of the heart muscle. M4 receptor subunit is abundant within CNS.

Although the data obtained may not exactly correlate with the actual number of transcripts due to different sizes of amplicons, nevertheless, the data show that there is expression. For a more accurate number of transcripts, a standard curve can be included in future experiments to avoid misinterpretation of results. It is also important to note that the expression does not indicate the translation of transcripts and the formation of a full-fledged receptor; nevertheless, the data obtained provide a primary picture of expression in cells.

Overall, the knowledge gained in this study could be further used in subsequent studies addressing functionality of cholinergic system within pituitary cells. Especially in studies that utilize RC-4BC cells.

## Supporting information

S1 Raw image (TIF)
