# Supplementary figures and images for: RC-4BC cells express nicotinic and muscarinic acetylcholine receptors
Source: PLoS One. 2022 Dec 16;17(12):e0279284. doi: 10.1371/journal.pone.0279284 (PMC9757584; doi:10.1371/journal.pone.0279284)

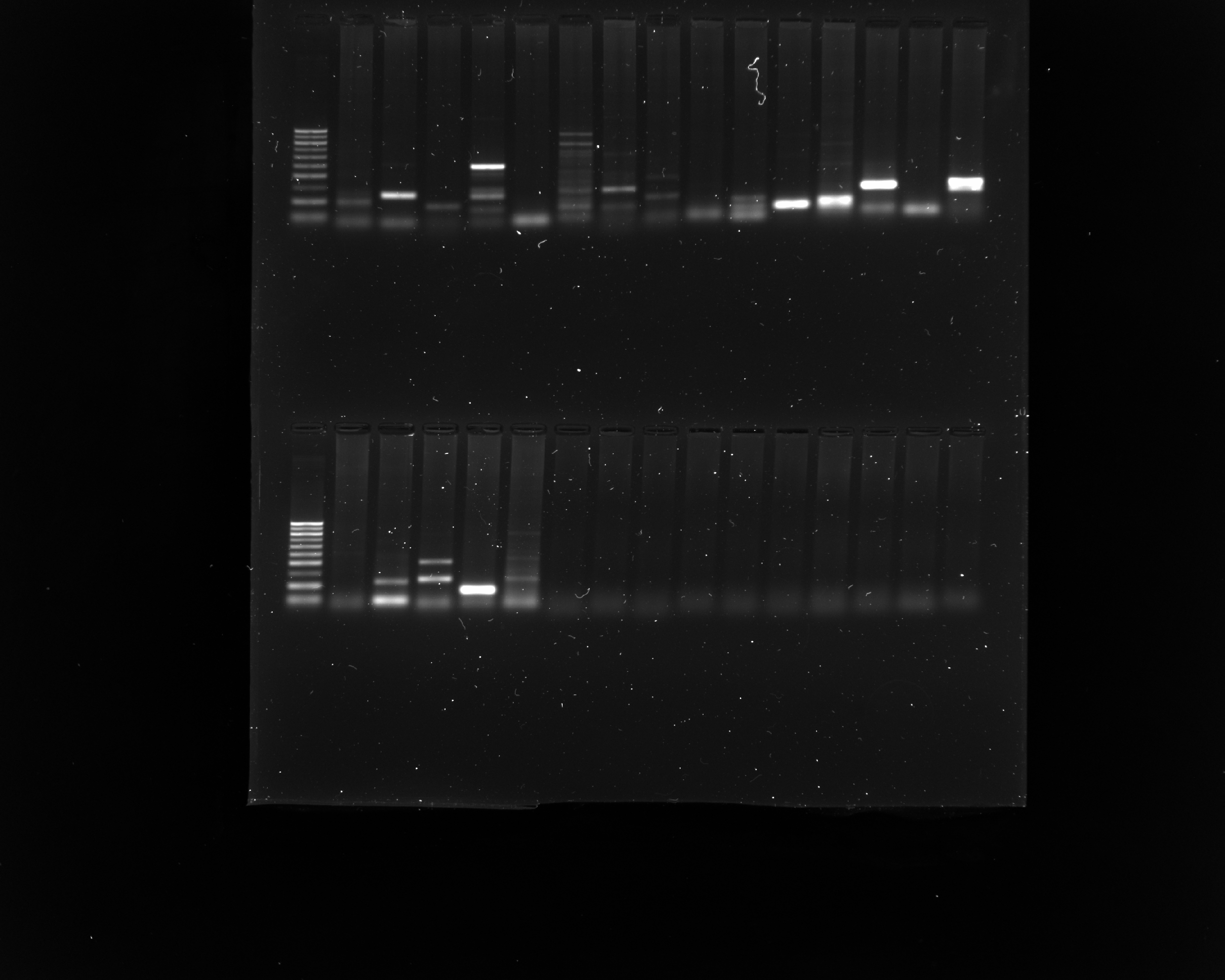

Supplement: S1 Raw image — (TIF) [file pone.0279284.s001.tif]
